# Ultrasound findings of fibroepithelial polyp in the fetal bladder: a case report

**DOI:** 10.3389/fonc.2024.1484918

**Published:** 2024-12-11

**Authors:** Xin Chen, Hong Luo

**Affiliations:** ^1^ Department of Diagnostic Ultrasound, West China Second University Hospital, Sichuan University, Chengdu, China; ^2^ Key Laboratory of Obstetric & Gynecologic and Pediatric Diseases and Birth Defects of Ministry of Education, Chengdu, China

**Keywords:** fibroepithelial polyp, prenatal diagnosis, ultrasound, benign tumor, fetal bladder

## Abstract

Fibroepithelial polyps are rare benign tumors originating from the mesoderm and are more commonly found in the renal pelvis and distal ureter and less frequently in the proximal ureter or bladder. This case report presents a fibroepithelial polyp occurring in the bladder of the fetus, showcasing its two-dimensional ultrasound, three-dimensional ultrasound, color Doppler, and spectral Doppler ultrasound findings, providing a reference for the accurate diagnosis of this condition.

## 1 Introduction

Fibroepithelial polyps (FEPs) are rare benign tumors originating from the mesoderm and are more commonly found in the renal pelvis and distal ureter and less frequently in the proximal ureter or bladder (1). This case report describes a fibroepithelial polyp occurring in the fetal bladder, analyzing its prenatal and postnatal ultrasound findings as well as prognosis.

## 2 Case report

**Figure 1:**
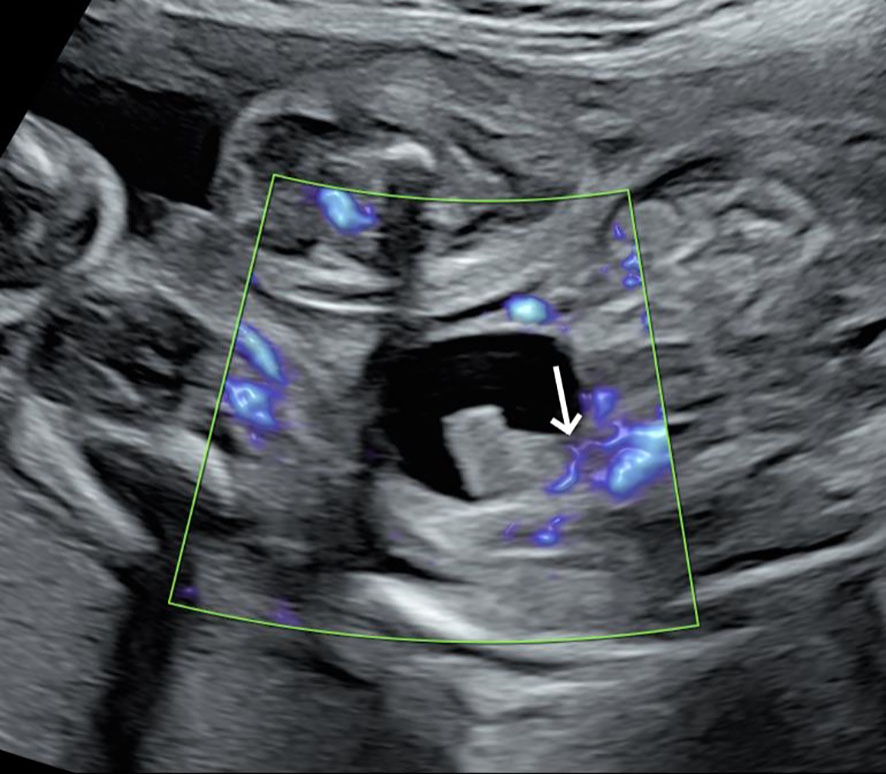
At 26 weeks of gestation, a hypoechoic mass is observed protruding from the posterior wall of the fetal bladder into the bladder cavity, with slow flow revealing minimal blood flow signals at the base of the mass.

**Figure 2:**
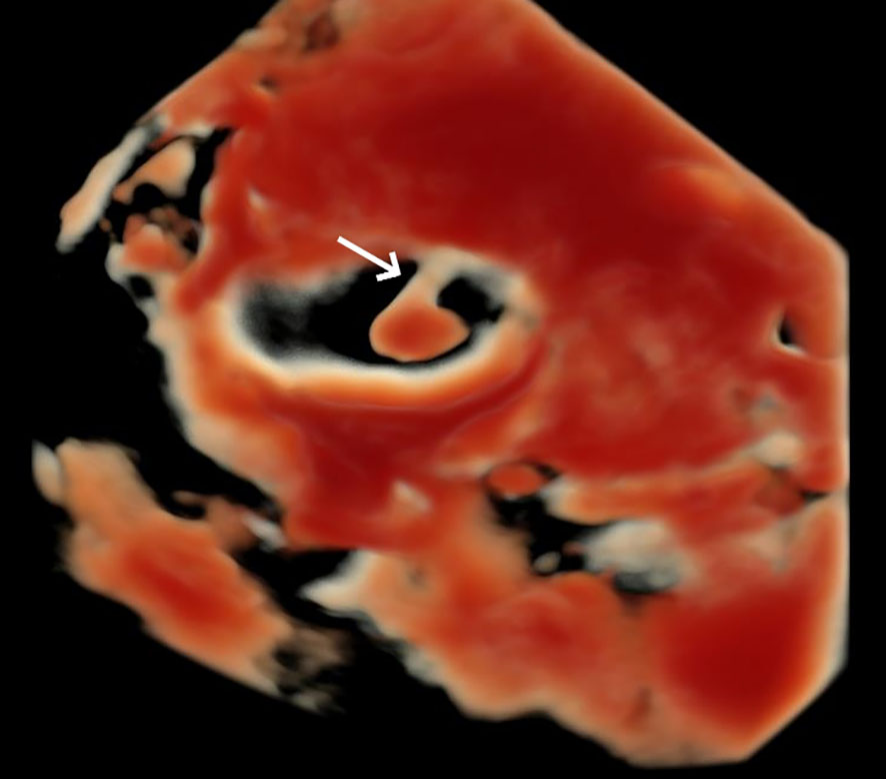
Three-dimensional ultrasound surface imaging in HD-LIVE mode shows the mass with a relatively regular shape, connected to the posterior bladder wall by a stalk-like echo (arrow).

**Figure 3:**
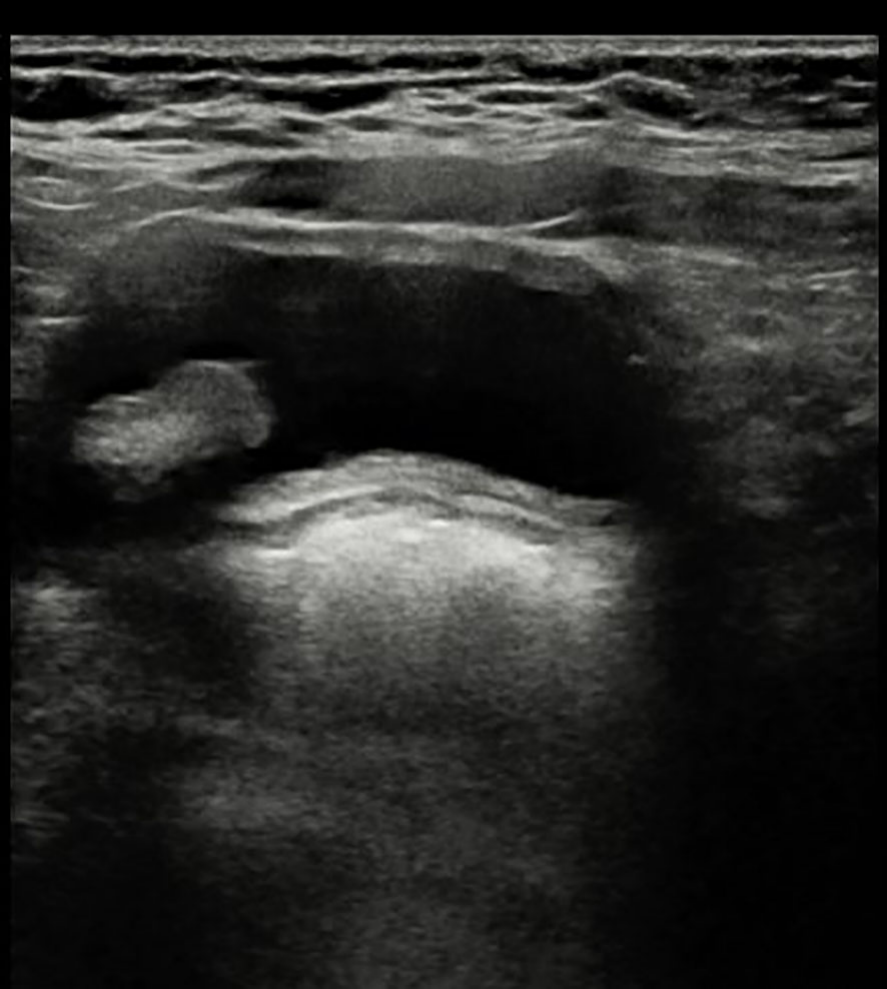
Follow-up examination at 10+ months after birth shows a hypoechoic mass located on the right side of the bladder, elongated in shape with uneven internal echoes and areas of increased echogenicity.

**Figure 4:**
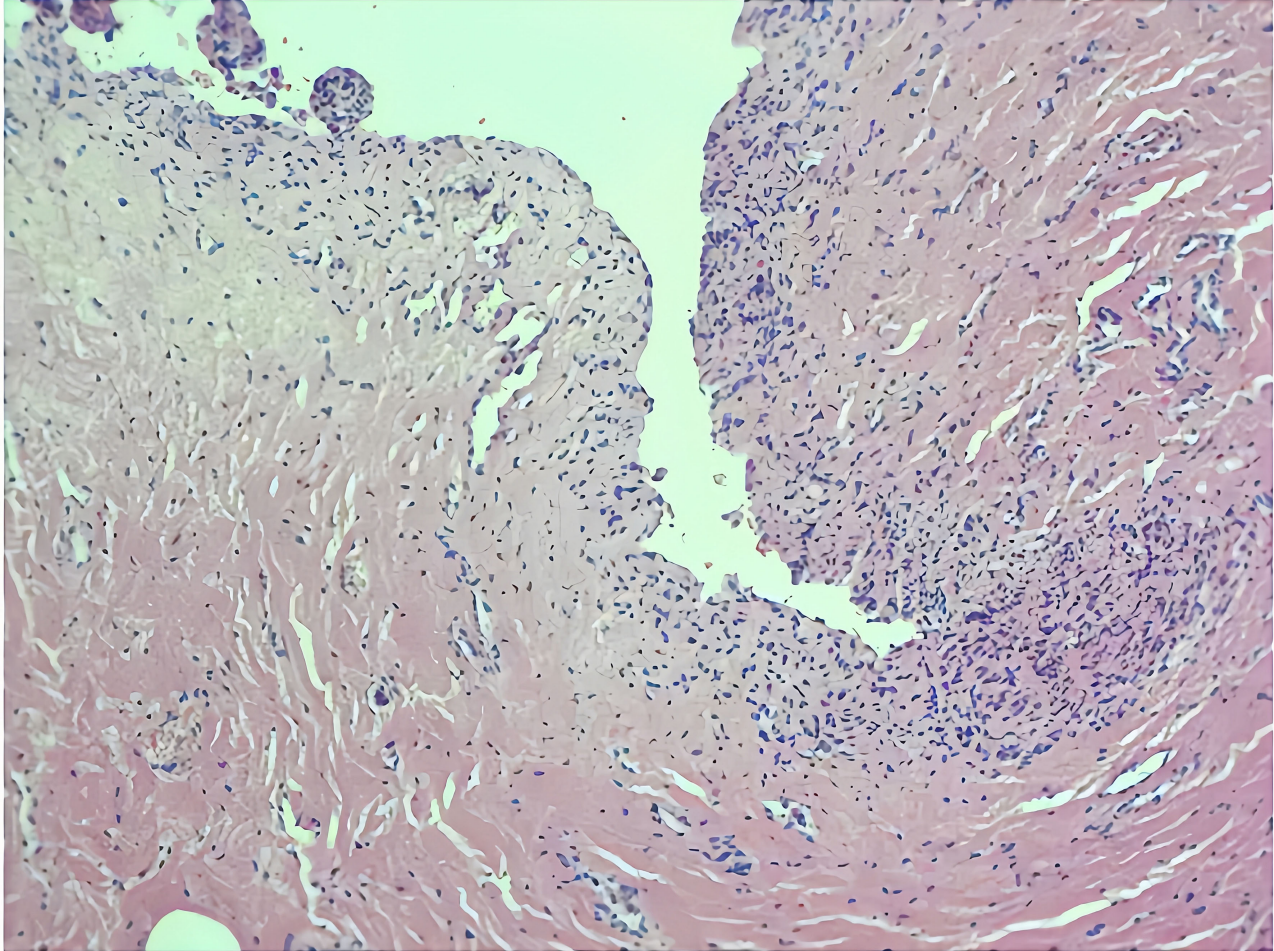
Postoperative pathology confirms the diagnosis of fibroepithelial polyp.

A 31-year-old pregnant woman, G^0^P^0^, presented to our hospital due to the discovery of a fetal bladder mass at an outside facility. A targeted ultrasound examination at our hospital revealed a hypoechoic mass measuring 1.2 × 0.6 × 1.3 cm, located adjacent to the posterior wall of the fetal bladder. The mass had a relatively regular shape and exhibited homogeneous internal echogenicity. Slow flow imaging showed a small amount of blood flow signal at the base of the mass (**Figure 1**), with a resistive index (RI) of 0.78. Three-dimensional ultrasound showed a thin, stalk-like echo connecting the weak echoic mass to the posterior wall of the bladder (**Figure 2**), with a relatively smooth bladder wall. MRI examination showed an abnormal signal in the form of a thick strip protruding from the posterior wall of the bladder into the bladder, with an undetermined nature. The patient was followed up with an ultrasound every 2–4 weeks. Prior to delivery at 40 weeks, the examination revealed that the mass had enlarged to 1.2 × 1.0 × 1.4 cm, with uneven internal echoes and an irregular shape. Chromosome examination showed no abnormalities in the fetus. The pregnant woman gave birth to a male baby at 40 weeks of gestation, with a birth weight of 3,030 g. After birth, urination was normal, and regular ultrasound examinations of the urinary system revealed an elongated hypoechoic structure located slightly to the right side of the bladder neck, measuring approximately 0.8 × 0.7 × 1.2 cm. The shape appeared somewhat irregular with relatively clear margins. Within it, a hyperechoic structure of approximately 0.4 cm in diameter was visible, accompanied by posterior acoustic shadowing (**Figure 3**). No obvious blood flow signals were detected within the mass, and the hypoechoic structure was contiguous with the posterior wall of the bladder. The infant underwent cystoscopy, transurethral bladder tumor resection, and bladder cystostomy at 10 months of age, during which a tumor measuring approximately 1.6 × 1.4 × 1.2 cm in size was found on the posterior wall of the bladder, with a tough texture and a stalk on the surface and was connected to the posterior wall of the bladder, approximately 1 cm away from the opening of the bilateral ureters. Pathological examination showed that the mass was a fibroepithelial polyp (**Figure 4**).

## 3 Discussion

FEPs in the urinary system can occur in any part of the urinary tract, including the renal pelvis, ureters, bladder, and urethra, with the most common occurrence in the posterior urethra. FEPs in the bladder are rare, and they are more prevalent in men than in women (2). The pathogenesis of FEPs in the bladder is not yet clear, but chronic infections, obstruction, trauma, or congenital factors may contribute to their development. Bladder FEPs are usually seen in infants and children, with few reported cases in adults; literature searches reveal no reports from the fetal period. The clinical symptoms associated with bladder FEPs are related to the site and size of the mass, primarily manifesting as hematuria, urinary tract infections, and difficulties in urination (3). Ultrasound has become the preferred diagnostic method for bladder FEPs due to its non-invasive nature, convenience, and high reproducibility. Other diagnostic methods include CT, MRI, cystourethrography, and cystoscopy. The characteristic ultrasound appearance features a solid echo mass connected to the bladder wall through a thin stalk, with clear boundaries, poor internal blood flow, and no signs of invasion into the bladder wall. Differentiation from other malignant tumors in the bladder, such as the most common pediatric rhabdomyosarcoma, is necessary (4). Bladder rhabdomyosarcoma typically occurs in the trigonal area of the bladder, often presenting with infiltrative growth resembling a grape-like mass. It usually lacks a stalk or has a wide-based stalk, with a larger volume and richer blood flow compared to FEPs. There is a certain probability that FEPs in the bladder may undergo squamous metaplasia and further develop into urothelial carcinoma; once detected, surgical removal is recommended. Surgical methods include traditional open surgery or transurethral resection of bladder polyps, the latter of which has the advantages of being less invasive and promoting quicker recovery, making it the preferred approach. The probability of recurrence after removal of FEPs is low, but long-term follow-up observation is still necessary (5, 6).

## Data Availability

The raw data supporting the conclusions of this article will be made available by the authors, without undue reservation.

## References

[1] Keçeli AM Dönmez Mİ Kılınç ANU. Fibroepithelial polyp at the bladder neck presenting with gross hematuria in a 5-year-old boy. J Endourology Case Rep. (2020) 6:107–9. doi: 10.1089/cren.2019.0125 PMC7580593 33102701

[2] Rousseau S Peycelon M Grosos C Bidault V Poupalou A Martin G. Management of lower urinary tract fibroepithelial polyps in children. J Pediatr Surg. (2021) 56:332–6. doi: 10.1016/j.jpedsurg.2020.05.030 32641248

[3] Agarwal S Sharma D Pandey S Sankhwar S. Benign fibroepithelial bladder polyp: A rare cause of childhood haematuria. BMJ Case Rep. (2018). doi: 10.1136/bcr-2018-226050 PMC6119361 30150352

[4] Shelmerdine SC Lorenzo AJ Gupta AA Chavhan GB. Pearls and pitfalls in diagnosing pediatric urinary bladder masses. RadioGraphics. (2017) 37:1872–91. doi: 10.1148/rg.2017170031 29019749

[5] Zhu S He L Zheng C Hou Y. Bladder mulberry-like fibroepithelial polyp with calcification and squamous cell metaplasia mimicking bladder carcinoma: case report and literature review. J Int Med Res. (2020) 48:1–6. doi: 10.1177/0300060519896911 PMC7113808 32008408

[6] Jayman J Tourchi A Shabaninia M Maruf M DiCarlo H Gearhart JP. The surgical management of bladder polyps in the setting of exstrophy epispadias complex. Urology. (2017) 109:171–4. doi: 10.1016/j.urology.2017.06.023 28652161

